# Reliability and structural validity of the Female Sexual Function Index in Spanish female university students

**DOI:** 10.3389/fpsyg.2026.1877071

**Published:** 2026-07-24

**Authors:** Laura Vicente-Gonzalez, F. Javier Del Río Olvera, Manuel A. García-Sedeño, Jose Luis Vicente-Villardon, Silvia V. Navarro-Murcia, Yolanda Medina-Mesa, Amor Espinosa-García, Sonia Franco-Jaén

**Affiliations:** 1 Department of Statistics, Universidad de Salamanca, Salamanca, Spain; 2 Department of Psychology, University of Cádiz, Cádiz, Spain; 3 Department of Social Anthropology, Basic Psychology and Public Health, Universidad Pablo de Olavide, Seville, Spain

**Keywords:** confirmatory factor analysis, female sexual function, female university students, FSFI, questionnaire validation

## Abstract

**Introduction:**

The Female Sexual Function Index (FSFI) is a questionnaire designed to assess sexual response in women and is widely used in both research and clinical settings. A systematic review has questioned the reliability of the instrument. The aim of the present study was to examine the reliability and factor structure of the questionnaire in Spanish female university students.

**Methodology:**

The sample consisted of 263 female university students, with a mean age of 22.67 years (SD = 6.08). The original 19-item version of the Female Sexual Function Index was used. The questionnaire assesses six different domains: Sexual desire, arousal, lubrication, orgasm, satisfaction, and pain.

**Results:**

Item analysis showed that item–total correlations ranged from 0.498 to 0.921. The reliability of the questionnaire, assessed using Cronbach’s alpha, was 0.975, while the reliability coefficients for each of the scales ranged from 0.803 to 0.972. The statistical indicators used to assess the suitability of conducting factor analysis were satisfactory. Finally, the confirmatory factor analysis indices indicated a good fit of the questionnaire to the six-factor structure of the original model.

**Discussion:**

The results indicate that the FSFI demonstrates good psychometric properties, supporting its use in both research and clinical settings. Future studies are recommended to replicate the validation to verify the continued validity of the instrument, as well as its adaptation to the updated criteria of the main diagnostic manuals.

## Introduction

Sexual health, defined as a state of physical, emotional, mental, and social well-being in relation to sexuality, and not merely the absence of disease, dysfunction, or disability (World Health Organization (WHO), 2002), constitutes an essential component of health in women. Sexual health is associated not only with the absence of illness and the presence of well-being, but also with the frequency of sexual activity. Previous studies have shown that sexual inactivity is associated with a higher prevalence of cancer, bladder disorders, major surgery, visual impairment, mental health conditions, cardiovascular disease, diabetes, hypertension and hypercholesterolemia, hearing loss, dementia, psychological problems, dermatological conditions, musculoskeletal problems affecting the joints, bones, or back, gastrointestinal disorders, alcohol abuse, chronic wound care, and periodontal disease (Bach et al., 2013; Boyacıoğlu et al., 2023; Christensen et al., 2011; Dilixiati et al., 2024; Gök et al., 2023; Gupta et al., 2011; Karakose et al., 2023; Khorshidi et al., 2022; Miner et al., 2012; Oveisi et al., 2022; Palacios-Ceña et al., 2012). A recent study reported that life satisfaction is positively associated with female sexual functioning (Bahrami et al., 2023). Furthermore, a systematic review found significant associations between positive indicators of sexual health and lower levels of depression and anxiety, as well as higher quality of life and greater life satisfaction in both women and men (Vasconcelos et al., 2024). Despite the existing literature suggesting that sexual health positively influences health-related outcomes, such as cardiovascular health (Gianotten, 2023), there may be exceptional circumstances. For example, a survey conducted in the general population during the COVID-19 lockdown restrictions found that sexual dysfunction was not significantly associated with participants’ quality of life (Chatterjee et al., 2022).

Currently, the DSM-5-TR (American Psychiatric Association (APA), 2024) classifies female sexual dysfunctions into female orgasmic disorder, female sexual interest/arousal disorder, and genito-pelvic pain/penetration disorder. In addition, other conditions that may also affect women include substance/medication-induced sexual dysfunction, another specified sexual dysfunction, and unspecified sexual dysfunction.

The prevalence of female sexual dysfunctions is high. According to some authors, it ranges between 19 and 50%, in a non-clinical American population (Jones, 2002), whereas other studies report prevalence rates between 40 and 50%, in a non-clinical Chilean population, and in Spanish women diagnosed with pelvic floor disorders (Quintana et al., 2025; Sánchez-Sánchez et al., 2020), and some findings even suggest rates as high as 98.5% in middle-aged Latin American women (Blümel et al., 2009). Prevalence estimates reported across studies vary considerably. However, a recent systematic review examining studies published between 2015 and 2024 in healthy women of reproductive age found a pooled prevalence of female sexual dysfunction of 47.81% (Heshmatnia et al., 2025), suggesting that this figure may represent a more reliable estimate of prevalence. The causes of female sexual dysfunctions may be organic or psychological in nature (del Río et al., 2020, 2018b). Despite their high prevalence, sexual dysfunctions in women continue to be underdiagnosed and undertreated (Arrom et al., 2021).

There are numerous assessment instruments for female sexual dysfunctions (Sierra et al., 2014), one of the most widely used being the Female Sexual Function Index (FSFI) (Rosen et al., 2000). As noted by Vallejo-Medina et al. (2018), the questionnaire has been translated and validated in more than 10 languages and across different populations, including middle-aged Brazilian women (Dall’Agno et al., 2019), premenopausal (Ahmed et al., 2017) and postmenopausal women (Pérez-Herrezuelo et al., 2019), pregnant women (Chang et al., 2009), and women with different types of cancer (Eaton et al., 2017; Jing et al., 2018; Liu et al., 2016). The use of a questionnaire that has been widely employed in previous research offers several advantages. First, it facilitates direct comparisons across studies conducted in different countries and populations. Second, the use of a common instrument increases confidence that the same construct is being assessed across investigations. In addition, it saves time and resources by avoiding the need to develop a new measure for an already established construct. Finally, the use of a previously validated instrument contributes to the stability of its psychometric properties and helps reduce measurement bias.

Several validation studies of the FSFI have been conducted in Spanish populations. Sánchez-Sánchez et al. (2020) performed a cultural adaptation and psychometric validation in women with pelvic floor disorders, reporting an overall reliability coefficient of 0.85 and identifying a six-factor structure through confirmatory factor analysis. Similarly, Arrom et al. (2021) validated a shortened 14-item version in Spanish women attending a gynecology clinic, reporting a reliability coefficient of 0.91 while retaining the six-factor structure of the original instrument. In addition, Pérez-Herrezuelo et al. (2019) validated the FSFI in postmenopausal women, obtaining a reliability coefficient of 0.964 and proposing a three-factor structure. In the Spanish validation conducted in 2008 (Fernández et al., 2008), the factorial structure did not fit the original questionnaire, showing a better fit for a three-factor model and obtaining a Cronbach’s alpha coefficient of 0.92. Rincón-Hernández et al. (2021) examined the temporal stability and clinical validity of the Spanish version in a Colombian population by assessing test–retest reliability and comparing a clinical group with a control group. Their findings supported the adequacy of the questionnaire for the assessment of female sexual dysfunction according to the DSM-5. Sánchez-Sánchez et al. (2020) conducted a cultural adaptation and validation study in a sample of 323 Spanish women and concluded that the questionnaire showed adequate psychometric properties for assessing female sexual function. Similar findings were reported by Quintana et al. (2025) in a sample of Chilean women and by Vallejo-Medina et al. (2018) in a sample of Colombian women. These validation studies were based on the original questionnaire developed by Rosen et al. (2000), generally retaining its initial six-factor structure, although some studies reported alternative factorial solutions (Fernández et al., 2008).

In a systematic review examining the psychometric properties of the Female Sexual Function Index (FSFI) (Neijenhuijs et al., 2019), which included 83 studies assessing the reliability and validity of the questionnaire across different populations and languages, the evidence regarding internal consistency was considered sufficient and of moderate quality, whereas the evidence concerning reliability was sufficient but of low quality. The authors highlighted that the lack of evidence for some of the psychometric properties of the FSFI underscores the importance of continuing to investigate the validity of these measures and recommended further examination of the factorial structure of the questionnaire. For this reason, the aim of the present study was to examine the reliability and factorial structure of the Female Sexual Function Index questionnaire and to examine the validity of its internal structure in a university population.

## Method

### Participants

The sample consisted of 263 female university students, with a mean age of 22.67 years (SD = 6.08). Sociodemographic data is presented in Table 1. The youngest participant was 18 years old and the oldest was 54 years old. Regarding sexual orientation, 46 women (17.49%) identified themselves as bisexual, 200 (76.05%) as heterosexual, 14 (5.32%) as homosexual, and 1 (0.38%) as asexual, while 2 participants (0.76%) preferred not to answer this question. Participants were asked whether they were currently in a relationship at the time of participation in the study. A total of 58.94% (155) reported having a partner, whereas 41.06% (108) reported not having a partner. Participants were also asked whether they engaged in regular physical exercise, and 69.58% (183) reported that they did, compared to 30.42% (80) who reported not engaging in regular exercise. In addition, participants were asked whether they used any contraceptive methods in their sexual relationships. A total of 63.12% (166) reported using contraceptive methods, whereas 36.88% (97) reported not using them. Participants were also asked whether they considered themselves religious, with 37.64% (99) responding affirmatively and 62.36% (164) responding negatively. Finally, participants were asked about their political ideology. A total of 53.61% (141) reported a left-wing ideology, 14.45% (38) identified as politically centrist, 9.13% (24) reported a right-wing ideology, and 22.81% (60) preferred not to answer this question. Inclusion criteria required participants to be at least 18 years of age, provide informed consent, and have engaged in sexual activity within the previous 12 months.

**Table 1:** Sociodemographic characteristics of the sample.

Sociodemographic information	*n*	%
Sexual orientation		
Bisexual	46	17.49
Heterosexual	200	76.05
Homosexual	14	5.32
Asexual	1	0.38
Declined to answer	2	0.76
Currently in a relationship		
In a relationship	155	58.94
Not in a relationship	108	41.06
Physical activity		
Regular physical activity	183	69.58
No regular physical activity	80	30.42
Use of contraceptive methods		
Uses contraceptive methods	166	63.12
Does not use contraceptive methods	97	36.88
Religiosity		
Religious	99	37.64
Non-religious	164	62.36
Political ideology		
Left-wing	141	53.61
Center	38	14.45
Right-wing	24	9.13
Declined to answer	60	22.81

*n*, number of participants; %, percentage.

### Instrument

An *ad hoc* questionnaire was used to collect sociodemographic data: age, sexual orientation, relationship status, physical activity, contraceptive use, religiosity, and political ideology.

The Female Sexual Function Index (FSFI) (Rosen et al., 2000) was used. In its original version, the questionnaire consists of 19 items answered on a 5-point (1 to 5, where a score of 1 may indicate “almost never or never” or “very low or none,” whereas a score of 5 may indicate “almost always or always” or “very high”) or 6-point (0 to 5, where a score of 0 indicates no sexual activity, whereas a score of 5 may indicate “almost always or always” or “very high”) Likert-type response scale. A total score below 26.5 may be indicative of problems in the sexual domain (Rosen et al., 2000). The instrument assesses six different domains: sexual desire (During the past 4 weeks, how often have you felt sexual desire or interest?), arousal (During the past 4 weeks, how often have you felt sexually aroused during sexual activity or intercourse—either alone or with a partner?), lubrication (During the past 4 weeks, how often have you become lubricated (“wet”) during sexual activity—either alone or with a partner?), orgasm (During the past 4 weeks, when you engaged in sexual stimulation or sexual activity—either alone or with a partner—how often did you achieve orgasm [“come”]?), satisfaction (During the past 4 weeks, how satisfied have you been with the level of emotional closeness during sexual activity with your partner?), and pain (During the past 4 weeks, how often have you experienced discomfort or pain following vaginal penetration?). For the present study, the 19-item Spanish version validated by Vallejo-Medina et al. (2018) was used.

### Procedure

The study followed an instrumental methodology (with the aim of developing assessment instruments and examining their psychometric properties), in accordance with the recommendations of the literature (del Río et al., 2018a). The sample was recruited at a Spanish public university using convenience sampling. Data was collected between September 2023 and January 2024. Data was collected through an online questionnaire accessed via a link (University of Cádiz Google Form) distributed by email. All data collected was anonymous.

### Data analysis

To analyze the psychometric properties of the FSFI, descriptive statistics and item reliability coefficients were calculated. The literature recommends removing items with corrected item–total correlations below 0.20 (Ebel, 1965). Internal consistency was assessed using Cronbach’s alpha and McDonald’s omega coefficients. Prior to factor analysis, the suitability of the data was evaluated using the Kaiser–Meyer–Olkin (KMO) measure and Bartlett’s test of sphericity. Once these assumptions had been verified, a confirmatory factor analysis was conducted. Model fit was evaluated using the RMSEA, CFI, TLI, and SRMR indices. The recommended cutoff values for these fit indices are RMSEA ≤ 0.08, CFI ≥ 0.90, TLI ≥ 0.90, and SRMR ≤ 0.08 (Hu and Bentler, 1999). Given the ordinal qualitative nature of the Likert-type response scales, a Logistic Biplot technique for ordinal data was employed (Hernández-Sánchez et al., 2025). This methodology allows for the simultaneous visual representation of participants and questionnaire items, facilitating the interpretation of the interrelationship structure among the dimensions assessed. Unlike classical dimensionality reduction methods, this approach adequately handles the cumulative probabilities of response categories, making it particularly suitable for the analysis of psychometric scales in which a gradation in the intensity of the measured construct is assumed. Finally, normality was assessed using the Kolmogorov–Smirnov test, and correlations were examined using Spearman’s rank-order correlation coefficient. All analyses were conducted using SPSS Version 24, R (R Core Team, 2022) and RStudio Version 2026.05.0 (Posit Team, 2025).

## Results

First, the items were analyzed to verify that they met the statistical criteria required for inclusion in the final version of the questionnaire, as items with item–total correlations below 0.20 are generally recommended for removal, whereas those with values between 0.20 and 0.30 should be considered for revision (Ebel, 1965). None of the items showed a corrected item–total correlation below 0.30; therefore, no item modification or removal was necessary (Table 2). Information for each subscale is also provided, apart from the Desire subscale, as it consists of only two items (Table 3).

**Table 2:** Item mean and variance, corrected item–total correlation, and questionnaire alpha if the item is deleted, for the scale as a whole.

Items	*M*	*V*	*r*_i-T_	*α*
Item 1	3.411	0.942	0.498	0.976
Item 2	3.468	0.812	0.503	0.976
Item 3	3.567	3.318	0.886	0.972
Item 4	3.354	3.004	0.905	0.972
Item 5	3.506	3.208	0.921	0.972
Item 6	3.464	3.359	0.908	0.972
Item 7	3.479	3.497	0.885	0.972
Item 8	3.665	3.363	0.909	0.972
Item 9	3.418	3.422	0.903	0.972
Item 10	3.639	3.379	0.906	0.972
Item 11	3.308	3.430	0.825	0.973
Item 12	3.335	3.166	0.811	0.973
Item 13	3.426	3.476	0.856	0.973
Item 14	3.525	4.036	0.885	0.972
Item 15	4.008	1.483	0.658	0.975
Item 16	3.848	1.467	0.630	0.975
Item 17	3.304	3.702	0.805	0.973
Item 18	3.452	3.845	0.805	0.973
Item 19	3.240	3.688	0.770	0.974

*M*, mean of the item; *V*, variance of the item; *r*_i-T_, corrected item–total correlation; *α*, questionnaire alpha if the item is deleted.

**Table 3:** Item mean and variance, corrected item–total correlation, and scale alpha if the item is deleted, item mean and variance within each subscale.

Subscales	*M*	*V*	*r*_i-T_	*α*
Arousal				
3	3.57	1.825	0.912	0.964
4	3.35	1.737	0.929	0.959
5	3.51	1.795	0.935	0.957
6	3.46	1.836	0.922	0.961
Lubrication				
7	3.48	1.874	0.902	0.970
8	3.67	1.837	0.939	0.960
9	3.42	1.853	0.933	0.961
10	3.64	1.842	0.941	0.959
Orgasm				
11	3.31	1.856	0.920	0.918
12	3.33	1.783	0.903	0.931
13	3.43	1.868	0.882	0.946
Satisfaction				
14	3.52	2.013	0.672	0.812
15	4.01	1.220	0.766	0.658
16	3.85	1.214	0.655	0.752
Pain				
17	3.30	1.928	0.923	0.943
18	3.45	1.965	0.931	0.938
19	3.24	1.924	0.908	0.955

*M*, mean of the item; *V*, variance of the item; *r*_i-T_, corrected item–total correlation; *α*, scales alpha if the item is deleted.

**Figure 1:**
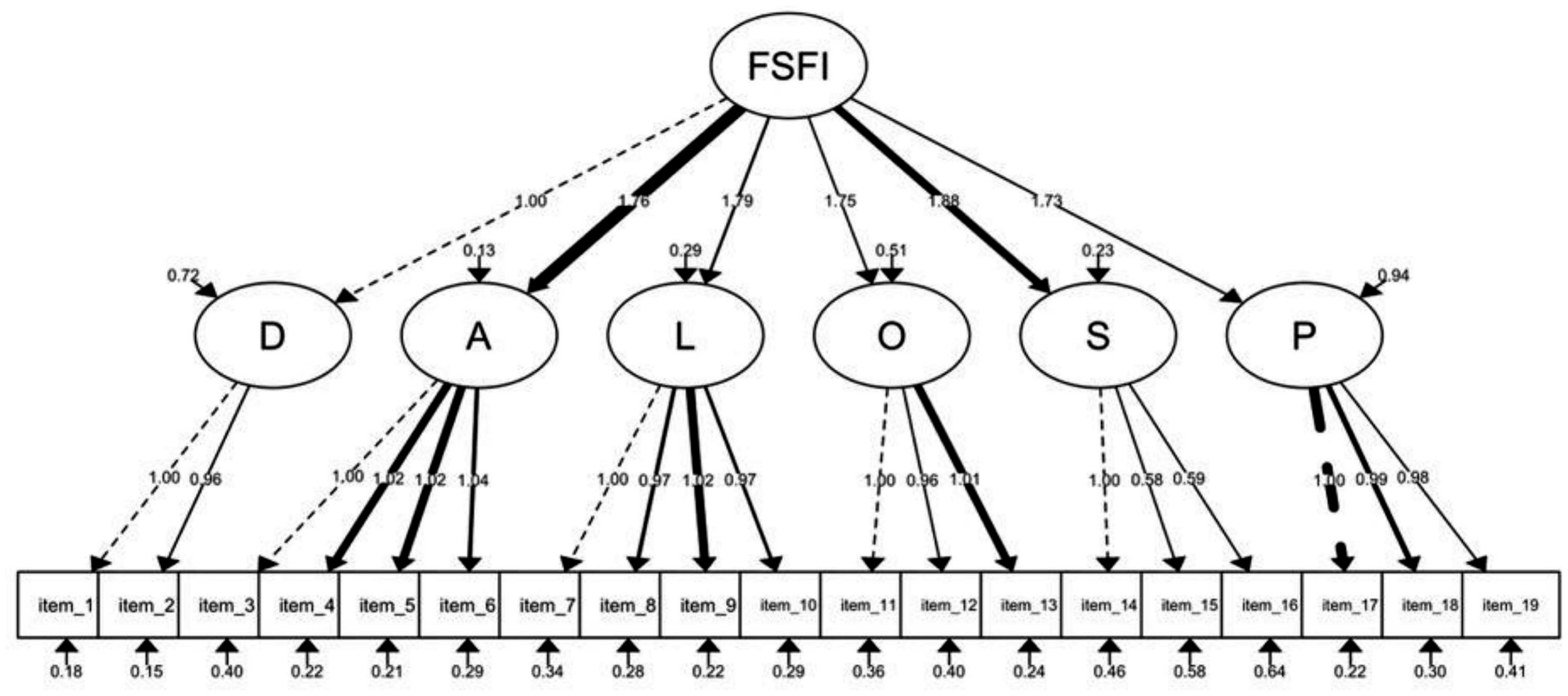
Diagram of the confirmatory factor analysis. FSFI, Female Sexual Function Index; D, desire; A, arousal; L, lubrication; O, orgasm; S, satisfaction; P, pain.

To verify the suitability criteria for conducting factor analysis, the Kaiser–Meyer–Olkin test (KMO = 0.95) and Bartlett’s test of sphericity (6958.40, *p* < 0.01) were performed. In all cases, the results confirmed the appropriateness of conducting factor analysis. The confirmatory factor analysis, according to the original factorial structure proposed by Rosen et al. (2000), showed good model fit indices (RMSEA = 0.072; CFI = 0.990; TLI = 0.989; SRMR = 0.012; *χ*^2^ = 296.33, degrees of freedom = 146), as shown in Figure 1.

Next, the reliability of the questionnaire and of each of the scales composing the instrument was calculated using Cronbach’s alpha and McDonald’s Omega (Table 4). The questionnaire is composed of six scales. The Desire scale includes items 1 and 2; the Arousal scale includes items 3, 4, 5, and 6; the Lubrication scale includes items 7, 8, 9, and 10; the Orgasm scale includes items 11, 12, and 13; the Satisfaction scale includes items 14, 15, and 16; and, finally, the Pain scale includes items 17, 18, and 19. The results exceeded the recommendations of Nunnally and Bernstein (1994) regarding the minimum reference values required for questionnaires used in research (Cronbach’s alpha values above 0.70). Therefore, the instrument demonstrated sufficient reliability values for use in the present study.

**Table 4:** Cronbach’s alpha coefficients and McDonald’s Omega of the questionnaire and the scales.

Subscales	*α*	Ω	*N*
Desire	0.763*	0.763*	2
Arousal	0.970	0.970	4
Lubrication	0.972	0.971	4
Orgasm	0.953	0.953	3
Satisfaction	0.803	0.846	3
Pain	0.963	0.963	3
Total	0.975	0.974	19

*α*, alpha coefficient; Ω, McDonald’s Omega; *N*, number of items; *, inter-item correlation coefficient.

**Figure 2:**
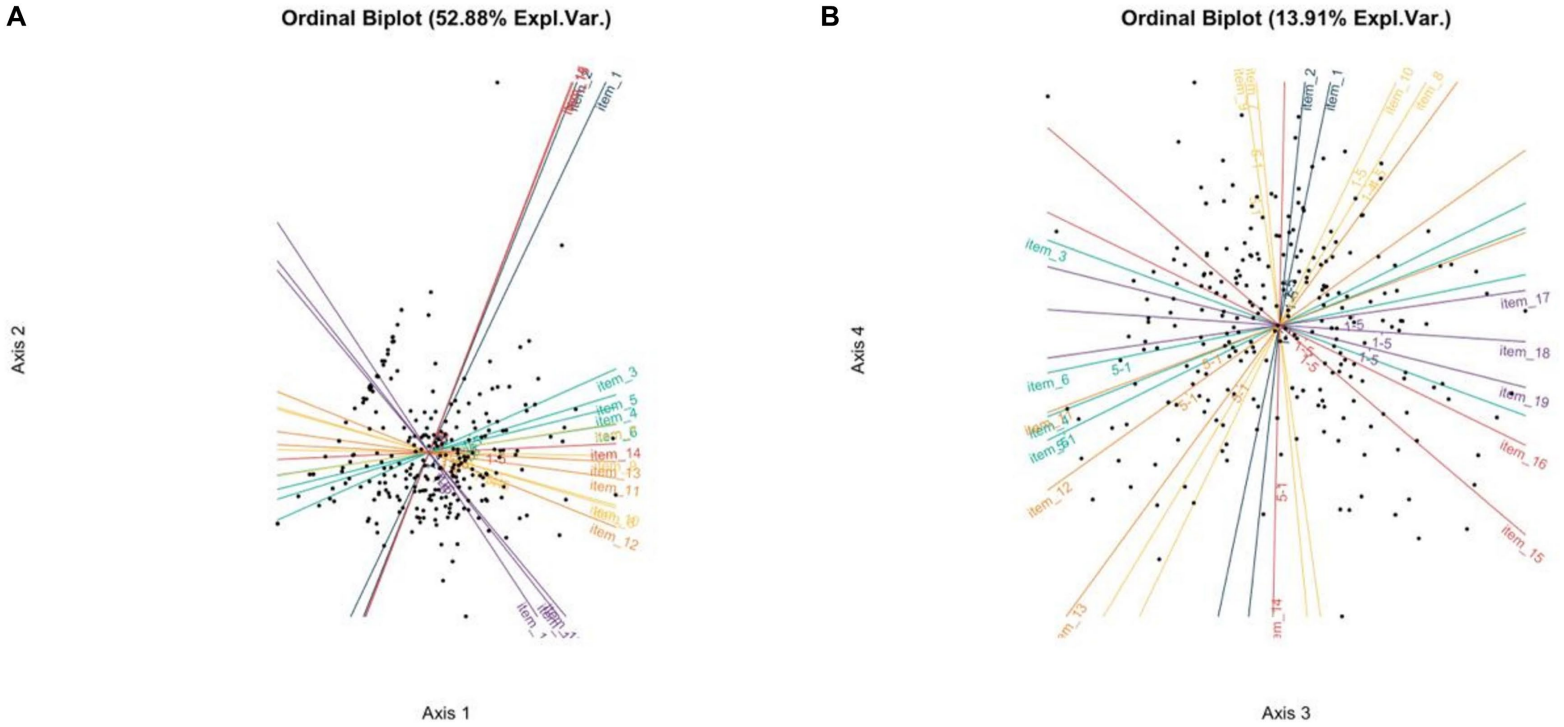
Ordinal logistic biplot of the FSFI items. **(A)** First factorial plane (dimensions 1 and 2), accounting for 52.88% of the explained variance (dimension 1 = 36.99%; Dimension 2 = 15.89%). **(B)** Second factorial plane (dimensions 3 and 4), accounting for 13.92% of the explained variance (dimension 3 = 7.01%; dimension 4 = 6.91%), resulting in a cumulative explained variance of 66.80%. Dots represent individual participants, and vectors represent item directions. Items are color-coded according to the original FSFI domains: desire (dark blue), arousal (green), lubrication (yellow/orange), orgasm (light brown), satisfaction (purple), and pain (red/dark brown).

To evaluate the internal structure and psychometric performance of the FSFI items while accounting for their ordinal nature, an Ordinal Logistic Biplot analysis (Figure 2) based on alternating gradient descent in cumulative probabilities was conducted (Hernández-Sánchez et al., 2025). The six-dimensional model showed an adequate fit to the data, with an Akaike Information Criterion (AIC) of −16235.73, a Bayesian Information Criterion (BIC) of −15334.80, and a Global Percentage of Correct Classification (PCC) of 93.79%. At the item level, model fit was satisfactory, with Nagelkerke pseudo-*R*^2^ values ranging from 0.617 (Item 14) to 0.890 (Item 16), indicating that the model accounted for a substantial proportion of the variability in the Likert-type response categories.

The factor structure showed that Dimension 1 accounted for the largest proportion of explained variance (36.99%). Most items exhibited high positive discrimination slopes on this dimension (e.g., Item 06 = 1.66; Item 11 = 1.72), suggesting that it reflects a broad component of sexual functioning. Dimension 2 accounted for 15.89% of the explained variance. Together, Dimensions 1 and 2 formed the first factorial plane, explaining 52.88% of the total variance (Figure 2A). Within this plane, the items belonging to the Desire domain (Item 01 and Item 02) and some Satisfaction items (Item 15 and Item 16) showed positive loadings on Dimension 2, whereas the Pain items (Item 17, Item 18, and Item 19) showed negative loadings (e.g., Item 17 = −1.35; Item 19 = −1.49). This pattern indicates a differentiation between desire- and satisfaction-related indicators and pain-related indicators along the second dimension.

The second factorial plane (Dimensions 3 and 4) accounted for 13.92% of the explained variance (7.01 and 6.91%, respectively), resulting in a cumulative explained variance of 66.80% up to Dimension 4 (Figure 2B). Examination of the item distribution according to their original theoretical dimensions revealed a differentiated spatial configuration. The Desire items (dark blue) were primarily aligned along Dimension 4, whereas the Lubrication (yellow) and Arousal (green) items formed distinct clusters within the right quadrants. In contrast, the Orgasm (light brown) and Satisfaction (purple) items were located predominantly within the lower quadrants. This spatial distribution supports the presence of a multidimensional latent structure consistent with the original theoretical organization of the FSFI and provides additional evidence for the differentiation of its subscales within the studied population.

Following Hernández-Sánchez et al. (2025), the labels “1–5” indicate the ordering of the response categories. A “1–5” configuration shows that the probability of selecting higher Likert-type response categories increases along the direction of the vector.

Finally, correlations were calculated between the different scales of the questionnaire and the total score (Table 5). As the data did not fit a normal distribution (K–S, *p* < 0.001), correlations were performed using Spearman’s rho test. All correlations were significant at the 0.01 level. The correlations among the different factors were high, which may indicate the presence of a general factor or the possibility of reducing the number of factors in the questionnaire, as suggested by other authors (Fernández et al., 2008).

**Table 5:** Correlations between the scales and the total score of the questionnaire.

Subscales	Arousal	Lubrication	Orgasm	Satisfaction	Pain	Total
Desire	0.552**	0.469**	0.384**	0.510**	0.320**	0.636**
Arousal		0.728**	0.733**	0.760**	0.506**	0.873**
Lubrication			0.596**	0.659**	0.601**	0.803**
Orgasm				0.683**	0.479**	0.792**
Satisfaction					0.562**	0.854**
Pain						0.727**

**, <0.01.

## Discussion

This study presents the results of the item analysis, reliability assessment (both for the full questionnaire and for each individual scale), confirmatory factor analysis, and correlations among the scales. The main objective of this study was to provide evidence regarding the validity of the instrument’s internal structure for use in a university population. The findings obtained from the reliability analyses (Cronbach’s alpha and McDonald’s omega) and the examination of the questionnaire’s factor structure (through confirmatory factor analysis and biplot analysis) confirm its suitability for use in university populations.

Prior to factor analysis, the suitability of the data was assessed using the KMO measure and Bartlett’s test of sphericity. The results supported the appropriateness of conducting factor analysis. A confirmatory factor analysis was subsequently performed to examine the original questionnaire structure proposed by Rosen et al. (2000), and the findings supported the six-factor model. This remains one of the most debated aspects of the instrument, as not all validation studies have reported model fit indices (Eaton et al., 2017; Rincón-Hernández et al., 2021), and the evidence regarding its factorial structure has been inconsistent. While some authors have developed shortened versions of the questionnaire (Dall’Agno et al., 2019; Arrom et al., 2021), others have reported factor solutions that differ from the original six-factor model (Chang et al., 2009; Liu et al., 2016; Pérez-Herrezuelo et al., 2019). According to the criteria proposed by Neijenhuijs et al. (2019), the results of the present study would be classified as providing sufficient evidence for structural validity, with very good methodological quality.

The reliability of the questionnaire and its scales was assessed using Cronbach’s alpha, yielding values ranging from 0.803 to 0.972, indicating that the questionnaire demonstrates adequate reliability for use in both research and clinical assessment settings. Similar results have been reported in previous studies (Pérez-Herrezuelo et al., 2019; Quintana et al., 2025; Sánchez-Sánchez et al., 2020; Vallejo-Medina et al., 2018), including studies involving shortened versions of the Female Sexual Function Index (Dall’Agno et al., 2019; Arrom et al., 2021). Regarding the item analysis, all items met the minimum criteria established in the psychometric literature to be considered adequate indicators of the construct. The lowest corrected item–total correlations were observed for Items 1 (0.498) and 2 (0.503), which comprise the Desire subscale; however, these values remained well above the recommended threshold. Although the item–total correlations for these two items were lower than those reported in previous validation studies (Pérez-Herrezuelo et al., 2019; Vallejo-Medina et al., 2018), the remaining items displayed comparable or even higher values than those previously reported (Pérez-Herrezuelo et al., 2019; Vallejo-Medina et al., 2018). Arrom et al. (2021) conducted a study aimed at developing a shortened version of the questionnaire, in which items were selected based on their item–total correlations. Their findings suggested that Items 1 and 2 could be candidates for removal. However, because both items belong to the Desire subscale, the authors decided to retain Item 1 in the final version of the instrument. These findings suggest that Items 1 and 2 may represent the least robust indicators within the questionnaire and could therefore be considered candidates for future revision. Further research is needed to examine their performance across different populations and contexts. Nevertheless, their current psychometric properties remain satisfactory and support their retention in the instrument. Reliability is one of the aspects of the questionnaire that has generated the greatest difficulties in reviews examining the psychometric performance of the Female Sexual Function Index (Neijenhuijs et al., 2019). Several studies obtained ratings of insufficient reliability, either because the reliability coefficients were too low or because the methodological quality was considered doubtful or inadequate due to the index used for calculation or the small sample size. In the present study, following the same procedure used in the review conducted by Neijenhuijs et al. (2019), and considering the value of the reliability coefficient obtained, the questionnaire would be classified as showing sufficient reliability and adequate methodological quality.

Among the limitations of the questionnaire, several issues should be highlighted. First, the number of response options differs across items. Not all items present the same number of response alternatives, which represent a methodological problem for statistical calculations and may complicate the use of data matrices. Standardizing the response options would therefore be of interest to avoid these methodological difficulties. In addition, the reliability of the questionnaire could be improved if all factors contained the same number of items, which would contribute to greater homogeneity among the factors. It is also important to note that updates in the major diagnostic manuals make it necessary to revise the scales of the questionnaire, as this may otherwise generate some degree of imprecision in diagnosis. It should be considered that the original questionnaire dates from 2000 (Rosen et al., 2000), whereas the major diagnostic manuals were updated in 2022 (World Health Organization (WHO), 2022 and 2024 (American Psychiatric Association (APA), 2024). Finally, considering the systematic review that motivated the present study (Neijenhuijs et al., 2019), it is necessary to continue evaluating this questionnaire according to the recommendations provided in that review to verify that the instrument continues to demonstrate adequate psychometric validity over time.

## Conclusion

The results indicate that the questionnaire demonstrates good psychometric properties, supporting the use of the Female Sexual Function Index in both research and clinical assessment protocols. The overall reliability of the questionnaire was 0.975 according to Cronbach’s alpha and 0.974 according to McDonald’s omega. These findings support the internal consistency of the questionnaire. Reliability coefficients for the individual dimensions ranged from 0.803 to 0.972 for Cronbach’s alpha and from 0.846 to 0.971 for McDonald’s omega. In addition, the data showed an adequate fit to the original six-factor structure of the questionnaire, which supports the construct validity of the questionnaire.

Future studies are recommended to replicate the validation to verify the continued validity of the instrument, as well as its adaptation to the updated criteria of the major diagnostic manuals.

## Data Availability

The datasets presented in this article are not readily available because the data has been anonymized and is only available to the corresponding author. Requests to access the datasets should be directed to F. Javier Del Río Olvera, franciscojavier.delrio@uca.es.
